# Energy Efficient Control of Ultrafast Spin Current to Induce Single Femtosecond Pulse Switching of a Ferromagnet

**DOI:** 10.1002/advs.202001996

**Published:** 2020-10-15

**Authors:** Quentin Remy, Junta Igarashi, Satoshi Iihama, Grégory Malinowski, Michel Hehn, Jon Gorchon, Julius Hohlfeld, Shunsuke Fukami, Hideo Ohno, Stéphane Mangin

**Affiliations:** ^1^ Université de Lorraine Institut Jean Lamour UMR CNRS Nancy 7198 France; ^2^ Laboratory for Nanoelectronics and Spintronics Research Institute of Electrical Communication Tohoku University 2‐1‐1 Katahira, Aoba Sendai 980‐8577 Japan; ^3^ WPI Advanced Institute for Materials Research Tohoku University 2‐1‐1 Katahira, Aoba Sendai 980‐8577 Japan; ^4^ Frontier Research Institute for Interdisciplinary Sciences Tohoku University 6‐3 Aramaki Aza Aoba Sendai 980‐8578 Japan; ^5^ Center for Science and Innovation in Spintronics Tohoku University 2‐1‐1 Katahira, Aoba Sendai 980‐8577 Japan; ^6^ Center for Spintronics Research Network Tohoku University 2‐1‐1 Katahira, Aoba Sendai 980‐8577 Japan; ^7^ Center for Innovative Integrated Electronic Systems Tohoku University 468‐1 Aramaki Aza Aoba Sendai 980‐0845 Japan

**Keywords:** femtosecond laser, magnetism, single shot all optical switching, spintronics

## Abstract

New methods to induce magnetization switching in a thin ferromagnetic material using femtosecond laser pulses without the assistance of an applied external magnetic field have recently attracted a lot of interest. It has been shown that by optically triggering the reversal of the magnetization in a GdFeCo layer, the magnetization of a nearby ferromagnetic thin film can also be reversed via spin currents originating in the GdFeCo layer. Here, using a similar structure, it is shown that the magnetization reversal of the GdFeCo is not required in order to reverse the magnetization of the ferromagnetic thin film. This switching is attributed to the ultrafast spin current and can be generated by the GdFeCo demagnetization. A larger energy efficiency of the ferromagnetic layer single pulse switching is obtained for a GdFeCo with a larger Gd concentration. Those ultrafast and energy efficient switchings observed in such spintronic devices open a new path toward ultrafast and energy efficient magnetic memories.

## Graphical abstract

In GdFeCo/Cu/[Co/Pt] spin valve, the ultrafast demagnetization of the ferrimagnetic GdFeCo alloy, generated by a single femtosecond laser pulse, is shown to generate enough spin current to switch the magnetization of the ferromagnetic Co/Pt multilayer. By increasing the Gd concentration, the Co/Pt switching is found to be more energy efficient.

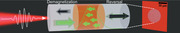

## 1 Introduction

All optical switching (AOS) of magnetization using ultrashort laser pulses, whether it is helicity dependent ^[^ ^1^, ^2^, ^3^, ^4^, ^5^, ^6^ ^]^ or helicity independent (AO‐HIS),^[^ ^4^, ^6^, ^7^, ^8^, ^9^, ^10^, ^11^, ^12^ ^]^ exhibits a very interesting out of equilibrium physics as well as promising practical implications.^[^ ^13^ ^]^ Many attempts to unveil the detailed mechanism of those phenomena have been carried out both theoretically^[^ ^8^, ^14^, ^15^, ^16^, ^17^, ^18^, ^19^, ^20^, ^21^, ^22^, ^23^, ^24^ ^]^ and experimentally.^[^ ^1^, ^2^, ^3^, ^4^, ^5^, ^6^, ^7^, ^8^, ^9^, ^10^, ^11^, ^12^, ^25^, ^26^, ^27^, ^28^ ^]^ AO‐HIS is observed after a single femtosecond laser pulse and most of the materials showing this effect are gadolinium‐based rare earth (RE)/transition metals (TMs) ferrimagnets.^[^ ^24^ ^]^ AO‐HIS has been extensively studied in amorphous GdFeCo alloy.^[^ ^10^ ^]^ It was shown that AO‐HIS could only be observed for a limited range of alloy concentration, laser fluence and pulse duration.^[^ ^10^, ^29^, ^30^ ^]^ The first models able to reproduce such behaviors considered two antiferromagnetically exchange coupled magnetization sublattices and different relaxation times for each sublattice.^[^ ^8^, ^14^, ^15^, ^16^, ^17^, ^24^ ^]^ However it was recently shown by Iihama et al.^[^ ^27^ ^]^ that AO‐HIS can be achieved in an uncoupled ferromagnetic layer within a spin valve structure.

Their experiment consisted in shooting a single laser pulse on a spin valve structure with a Gd_23.3_(FeCo)_76.7_ layer and a Co/Pt multilayer separated by 10 nm of Cu such that both magnetic layers are magnetically decoupled. It was shown that the Co/Pt multilayer magnetization could be reversed with a single pulse provided that the GdFeCo magnetization is switched (AO‐HIS) and that the Gd magnetization sublattice is initially oriented in the direction opposite to the magnetization of the Co/Pt multilayer. The ferromagnetic layer reversal is explained by the generation of a spin current due to the GdFeCo switching.

In the present paper, we first show that we can reproduce the Co/Pt layer switching with two different GdFeCo layer compositions. By changing the composition of the GdFeCo layer, we aim at tuning the polarization of the spin current and affect the single pulse switching of the ferromagnetic Co/Pt multilayer. We then demonstrate that the reversal of the ferromagnetic layer can be obtained without switching the GdFeCo magnetization. A partial demagnetization is sufficient. Furthermore, in that case the GdFeCo layer does not even exhibit AO‐HIS by itself because its Gd concentration is too high.^[^ ^10^ ^]^ Moreover, we show that the fluence threshold required to observe the ferromagnetic multilayer magnetization reversal is reduced by increasing the Gd concentration in the GdFeCo alloy.

## 2 Experimental Section

**Figure 1:**
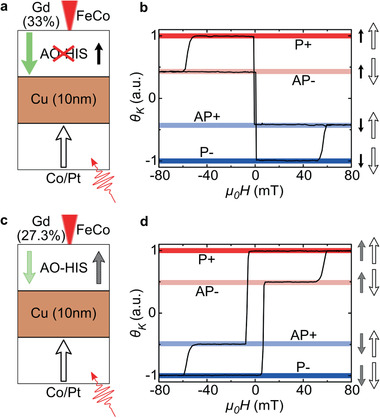
a) Simplified stack of the sample with *x* = 33% and b) its corresponding major hysteresis loop. c) Simplified stack of the sample with *x* = 27.3% and d) its corresponding major hysteresis loop. *θ*_Κ_ is the normalized Kerr rotation in arbitrary units. Green arrows indicate the Gd magnetization sublattice while black arrows indicate transition metals magnetizations. The red cone indicates where MOKE observation of magnetization (with a continuous laser or a MOKE microscope) is made. Black arrows on the hysteresis loops correspond to the black arrows in the simplified stacks.

Two spin valves consisting of a ferrimagnetic GdFeCo layer and a ferromagnetic Co/Pt multilayer separated by a 10 nm thick Cu spacer were studied. In the GdFeCo amorphous alloy, the magnetization of the RE sublattice is exchange coupled antiferromagnetically with the magnetization of the TM sublattice. Depending on the relative concentrations the net magnetization of the alloy is aligned along the RE magnetization sublattice (RE dominant) or the TM magnetization sublattice (TM dominant). The samples are, namely, glass/Ta(5)/Pt(4)/[Co(0,4)/Pt(1)]*_x_*_2_/Co(0,4)/Cu(10)/Gd*_x_*(FeCo)_1‐_ *_x_*(5)/Cu(5)/Ta(5), with *x* = 27.3% (TM dominant) or 33% (RE dominant). Numbers in parenthesis are the layer thicknesses in nanometers. They were grown by DC/RF magnetron sputtering as described by Iihama et al.^[^ ^27^ ^]^ In addition, two glass/Ta(5)/Cu(5)/Gd*_x_*(FeCo)_1‐_ *_x_*(5)/Cu(5)/Ta(5) samples of same concentrations were grown under the same conditions, as a reference. Note that the GdFeCo alloys have the same interfaces in all the samples. In **Figure** **1**b,d, hysteresis loops were measured by polar magneto optical Kerr effect (MOKE) using a continuous He‐Ne laser with a wavelength of 633 nm. For both samples, both magnetic layers exhibit perpendicular magnetic anisotropy. Linearly polarized femtosecond laser pulses of around 35 fs and with a central wavelength of 800 nm (1.55 eV) were used. For all measurements, the gaussian beam diameter (evaluated with the 1/e² convention) was around 115 µm. Samples were observed with a table top differential polar MOKE microscope using an LED (center wavelength around 630 nm) as a light source. As depicted in Figure 1, laser pulses were sent through the substrate, i.e., bottom side, and the magnetic domains were imaged subsequently from the other side, i.e., top side. In the opposite configuration, where the laser pulses were sent on the top side, samples exhibited the same qualitative behavior (see Section S8, (equation) #(S1)). All measurements were performed at room temperature.

## 3 Results

In spin valve structures, we will define the four possible magnetic configurations as parallel (P+ and P−) or antiparallel (AP+ and AP−) if the magnetizations of the TM sublattices (FeCo sublattice in the GdFeCo alloy and the Co/Pt multilayer) are parallel or antiparallel, respectively. Magnetic configurations are called positive (P+ or AP+) or negative (P− or AP−) if the Co/Pt magnetization is along the positive field direction or the negative field direction, respectively. Figure 1a,c shows a sketch of both samples in the P+ configuration. For all the figures, the green arrows represent the Gd magnetization sublattice while black arrows represent the FeCo magnetization sublattice (at the top) or the Co/Pt multilayer (at the bottom). In Figure 1a, the green arrow of Gd is bigger than the black one of FeCo to emphasize the difference in magnetization for both sublattices in this case.

We first consider the magnetic properties of the GdFeCo single layers only. They both show perpendicular magnetic anisotropy. Because the magneto‐optical Kerr effect for the wavelength used is mainly sensitive to the TM magnetization and weakly sensitive to the RE magnetization,^[^ ^31^ ^]^ we expect two different hysteresis loops depending on whether the ferrimagnetic alloy is RE dominant or TM dominant (see Section S5, Supporting Information). When *x* = 33%, the RE dominant case, the hysteresis loop is thus inverted compared to what is usually observed for a ferromagnet.

The major hysteresis loops for the spin valve structures are presented in Figure 1b,d for *x* = 33% and *x* = 27.3%, respectively. In the spin valve structure with *x* = 33%, we can then identify the inverted hysteresis loop (with a smaller coercive field in Figure 1b) caused by the GdFeCo response while the other hysteresis loop (with a higher coercive field) corresponds to the response of the Co/Pt multilayer. In the spin valve structure with *x* = 27.3%, the response of the Co/Pt multilayer can be easily identified since it is the same as for the previous spin valve (Figure 1d). The hysteresis loop with the smaller coercive field in Figure 1d then corresponds to the GdFeCo response. We observe that, for both spin valves, the Kerr signal due to the GdFeCo response is greater than the Co/Pt multilayer response. This is because the 10 nm of Cu attenuate the Kerr signal of the Co/Pt. A color code, shown in Figure 1b,d, is assigned to each magnetic configuration (P+, AP+, AP−, and P−) and will be the same for MOKE images. One important point is that we could conclude that the coupling between the two layers, deduced from the shift of the minor loops, is less than the resolution of the Hall probe (0.1 mT) used to measure magnetic fields. We consider in the following that the coupling is zero. We could even show (see Section S7, Supporting Information) that, at high fluence when a multidomain state is reached, P and AP magnetic configurations seem to be observed with equal probability. Whereas if a coupling was present, it should favor one type of magnetic configuration (P or AP).

We now look at the response of all samples to a single laser pulse. The GdFeCo single layer sample with *x* = 27.3% exhibits AO‐HIS as observed by several groups,^[^ ^1^, ^4^, ^7^, ^8^ ^]^ however with *x* = 33% the GdFeCo single layer sample does not show AO‐HIS (see Section S5, Supporting Information). In that latter case, only multidomain patterns are observed after exciting the sample with a single 35 fs laser pulse. Xu et al.^[^ ^10^ ^]^ showed that if there is no magnetization reversal on a long time scale, it will also not happen on a short time scale.

**Figure 2:**
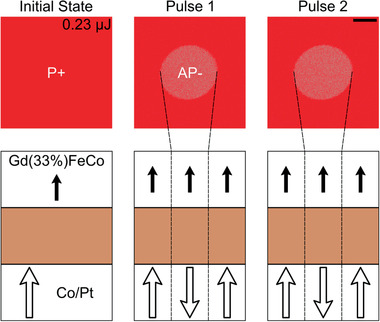
Results of sending a femtosecond laser pulse on the sample with *x* = 33% in a P+ configuration as observed using a MOKE microscope. The pulse energy is 0.23 µJ. The magnetic configuration is summarized below each image where only the transition metals magnetizations have been represented. The scale bar in the last image is 20 µm long.

Let us now concentrate on the spin valve structure. **Figure** **2** shows the result of irradiating single femtosecond laser pulses with an energy of 0.23 µJ on the spin valve sample with *x* = 33%. We first initialize the sample in a P+ configuration using an external magnetic field. After the first pulse, a light‐red color appears, corresponding to the reversal of the Co/Pt multilayer's magnetization. Exciting the sample with more pulses does not change the Kerr contrast. Performing the same experiment, with the same pulse energy, but starting in the AP− configuration, leaves the magnetic configuration unchanged (not shown). Simplified sketches of the spin valve magnetic configuration, showing only the magnetizations of the TM sublattice of each magnetic layer are displayed under each MOKE microscope image. Thus, we see that a single femtosecond laser pulse can induce the switching of the Co/Pt multilayer without affecting the GdFeCo orientation.

**Figure 3:**
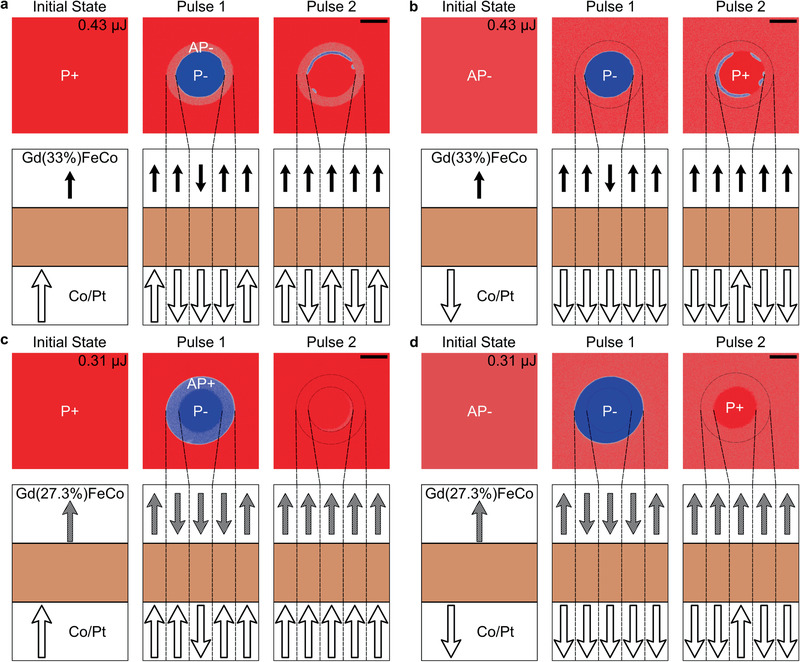
Results of sending a femtosecond laser pulse observed using a MOKE microscope. The magnetic configuration is summarized below each image where only the transition metals magnetizations have been represented. a,b) For the sample with *x* = 33% with a P+ and an AP− initial configuration, respectively. The pulse energy is 0.43 µJ. c,d) For the sample with *x* = 27.3% with a P+ and an AP− initial configuration, respectively. The pulse energy is 0.31 µJ. The scale bars in the last image of each figure are 30 µm long.

**Figure** **3** presents the results obtained for both samples while using pulses of higher energy. For the sample with *x* = 33% (Figure 3a,b) we observe the switching of both GdFeCo and Co/Pt magnetization after each pulse such that transitions P+ to P− and P− to P+ are observed but only in the central part of the laser spot. The clear red ring around this region corresponds to the reversal of the Co/Pt multilayer's magnetization, which only appears after the first pulse. This P+ to AP− transition is identical to the one observed at low fluence. In Figure 3b, one can see that no ring appears because the sample is already in the AP− configuration. Small AP+ domains at the rim appear after the second pulse that is attributed to the dipolar field that is stronger close to domain boundaries.

Figure 3c,d shows results obtained for the sample with *x* = 27.3%. Starting from a P+ configuration, we find two distinct behaviors. At a lower energy, i.e. what is observed further away from the center of the beam in Figure 3, only the GdFeCo layer reverses its magnetization. At higher energies, both the GdFeCo and the Co/Pt multilayer's magnetization reverse. When sending another laser pulse on the same spot, both magnetic domains disappear. We note that a tiny magnetic domain remains after the second pulse. In this region, the Co/Pt multilayer's magnetization has not been switched back, which can be explained by small deviations of the laser beam's position. Starting from an AP− configuration, only the GdFeCo layer's magnetization reverses after the first pulse while for the second pulse, the GdFeCo layer's magnetization is switched back and the Co/Pt multilayer's magnetization reverses in a smaller region.

**Figure 4:**
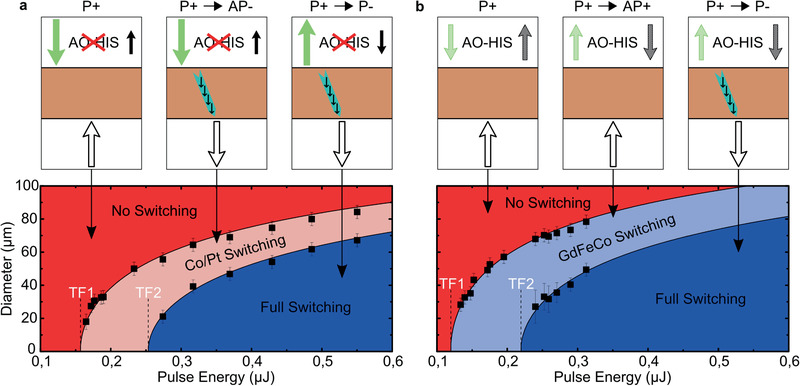
Domain size versus pulse energy diagrams for samples with a) *x* = 33% and b) *x* = 27.3% starting from the P+ configuration. TF1 and TF2 are the first and second threshold fluences, respectively. Sketches indicate what happens in each region of the diagram after the first laser pulse is sent. The turquoise arrow is meant to represent the spin current generated from the Gd demagnetization. Its polarization (the black arrows) is given by the initial Gd magnetization sublattice.

Figures 2 and 3 demonstrate the types of transitions which can be observed. To be more quantitative, we wish to determine the pulse energy required to observe one given transition. Because the laser beam has a gaussian profile, a smaller pulse energy implies that the created magnetic domain is smaller. However due to micromagnetic interactions, small reversed domains need to have a critical size to be stable on a long‐time scale and cannot be observed with our microscope setup.^[^ ^32^ ^]^ The typical minimum size we can observe in these samples is around 20 µm. In order to determine the threshold energy for a given transition, the size of the reversed magnetic domains as a function of the pulse energy is plotted as in **Figure** **4**. By fitting the data with a function parametrized by the threshold energy and the laser beam size, one can determine these threshold energies (see Section S4 of the Supporting Information for more details). Only the cases with a P+ configuration are shown. No difference in threshold fluences is found with the antiparallel case for a given sample, within uncertainties. All the threshold fluences are reported in **Table** **1**. No difference was found when starting from a P− or a P+ configuration, as expected from time reversal symmetry (not shown). In Figure 4, simplified stack sketches are shown to summarize what happens in each region. In both samples, nothing happens below the first threshold fluence (TF1) and both layers reverse their magnetization (i.e., P+ to P− transitions are always observed) above the second threshold fluence (TF2). However between TF1 and TF2, only the magnetization of the Co/Pt multilayer switches when *x* = 33% while when *x* = 27.3% only the GdFeCo layer's magnetization is reversed.

**Table 1:** Threshold fluences for the magnetization reversal of both magnetic layers in each sample. P and AP refer to the magnetic configuration before sending the first laser pulse

	Threshold fluences [mJ cm^−^²]			
*x*	GdFeCo (P)	Co/Pt (P)	GdFeCo (AP)	Co/Pt (AP)
27.3%	1.15 ± 0.07	2.11 ± 0.12	1.16 ± 0.07	No reversal
33%	2.66 ± 0.31	1.65 ± 0.19	2.72 ± 0.31	No reversal

## 4 Discussion

To explain those behaviors we have already excluded the possibility of an exchange coupling between both magnetic layers. We could also expect a magnetization switching induced by the dipolar field generated by one magnetic layer when it is demagnetized due to the local heating of the laser. In our structures with two magnetic layers, each layer generates a dipolar field which can impact its own magnetization (in that case we speak about demagnetizing field—case 1) or the other layer's magnetization (in that case we speak about stray field—case 2). Case 1 cannot explain the Co/Pt multilayers switching because it happens only starting from a P configuration. It is also ruled out for GdFeCo layers because AO‐HIS in single GdFeCo layers samples is not due to a dipolar field. The effect in case 2 is more subtle because GdFeCo may cross its compensation temperature when *x* = 33% (the compensation temperature is above room temperature when the sample is Gd dominant). Indeed, when crossing the compensation temperature the sign of the stray field can be inverted. However, we note that the dipolar field, if it has an effect, should be strongly dependent on the size of the magnetic domains as well as the way the sample is heated up. We notice the same reversal behavior for a wide range of fluences. In particular, the Co/Pt reversal in the sample with *x* = 33% is seen from 1.7 to 5.8 mJ cm^−^² (see Section S7 of the Supporting Information for the latter fluence), corresponding to various domain sizes and shapes. In addition, the same reversal behavior is observed when exciting from the top side whereas the light absorption profiles and therefore heating is quite different (see Section S6, Supporting Information). Thus, we believe that dipolar fields cannot explain the observed behavior. However, it can explain the multidomain patterns observed at high fluences together with thermal activation^[^ ^20^ ^]^ and it could partially explain the domains at the rim (where the dipolar field is the strongest) of the inner domains in Figure 3a,b.

Our explanation for the observed phenomena is based on i) the generation of a spin current due to the fast magnetization change of the Gd sublattice^[^ ^33^ ^]^ and ii) the possibility of this spin current to interact with the Co/Pt multilayer when it is sufficiently demagnetized as proposed by Iihama et al.^[^ ^27^ ^]^ Before diving into the details, we first summarize what is known regarding points (i) and (ii).

There is a growing awareness that nonlocal transfer of angular momentum is a key feature of AO‐HIS,^[^ ^25^ ^]^ which is related to the coupling between atomic spins of the ferrimagnet with conduction electrons.^[^ ^19^, ^21^, ^22^ ^]^ Therefore, conduction electrons can get spin polarized with a spin generation rate following the demagnetization rate.^[^ ^33^, ^34^ ^]^ This can be seen in models including an s–d interaction^[^ ^21^, ^35^, ^36^, ^37^ ^]^ where both magnetization sublattices contribute to the spin generation rate through their own demagnetization. This can lead to a spin current leaving the magnetic layer if it is sharing an interface with a metallic layer.^[^ ^38^, ^39^, ^40^, ^41^ ^]^ Choi and Min observed this spin current in RE/TM ferrimagnetic layers.^[^ ^33^ ^]^ The typical signal observed in these experiments shows two peaks of opposite signs at two different time delays. The first peak is mostly due to the ultrafast demagnetization of the TM sublattice which gives rise to a primary spin current whose polarization is given by the TM magnetization sublattice. The second peak is due to the continuing ultrafast demagnetization of the RE sublattice plus some spin dependent Seebeck effect.^[^ ^33^ ^]^ Thus, the secondary spin current has a polarization given by the RE magnetization sublattice. When the ferrimagnetic material contains Gd, Choi and Min^[^ ^33^ ^]^ showed that it is possible to increase the spin polarization of the current by increasing the amount of Gd. This can be understood by the fact that the total magnetic moment in the Gd sublattice increases and thus the Gd demagnetization rate will increase if the demagnetization time remains the same. This explains point (i).

Regarding the ferromagnetic layer, one needs to consider two different time scales. At short time scales (below ≈1 ps) the laser heating plus the heating due to hot carriers coming from other layers^[^ ^42^ ^]^ will cause the demagnetization of the Co/Pt multilayer. The primary spin current previously mentioned may also participate to the demagnetization.^[^ ^37^, ^43^ ^]^ In a second wave, the secondary spin current whose polarization is set by the Gd magnetization sublattice interacts with the ferromagnetic layer^[^ ^33^ ^]^ when the Co/Pt multilayer is demagnetized. We note that in the work of Bergeard et al.,^[^ ^42^ ^]^ the Co/Pt multilayer starts remagnetizing after 0.5 ps. However, increasing the fluence of the laser can extend the time range at which the ferromagnetic layer reaches its minimum magnetization.^[^ ^44^, ^45^ ^]^ Being demagnetized in the presence of a spin current, the ferromagnetic layer can remagnetize in the direction set by the polarization of the spin current. This is indeed consistent with the fact that, in Figure 2 when *x* = 33%, the Co/Pt multilayer's magnetization only switches when the Gd magnetization sublattice has the opposite direction. When *x* = 27.3%, the GdFeCo layer's magnetization reversal is assigned to AO‐HIS and the Co/Pt multilayer's magnetization reversal is due to its interaction with the spin current generated by the Gd magnetization sublattice. In Figure 4, we represent this spin current only when it is able to induce the reversal of the Co/Pt multilayer's magnetization. We can then see that the sample with *x* = 27.3% reproduces well the results of Iihama et al.^[^ ^27^ ^]^

The fact that the Co/Pt multilayer's magnetization can be reversed without observing the reversal of the GdFeCo layer's magnetization in the sample with *x* = 33% can be explained by previous works.^[^ ^21^, ^33^, ^34^ ^]^ Indeed, the key ingredient here is the spin current and the spin generation rate is proportional to the magnetization decay (−d*M*/d*t*). The reversal process of the GdFeCo layer's magnetization (when it exhibits AO‐HIS) is an ultrafast demagnetization followed by a slower reversal.^[^ ^7^ ^]^ The fastest dynamics happens during the demagnetization and thus no full magnetization reversal is needed to generate the spin current. We note that Choi and Min^[^ ^33^ ^]^ indeed measured the spin generation rate without reversing the GdFeCo magnetization. To observe the reversal of the ferromagnetic layer without switching the ferrimagnetic layer, it is then required to have a threshold fluence for AO‐HIS higher than a threshold fluence for a sufficient demagnetization of the Co/Pt multilayer. We demonstrate here that this can be done by increasing the Gd concentration, which not only increases the spin polarization of the current but also increases the threshold fluence required to observe AO‐HIS or even makes it impossible to observe anymore as in the case of *x* = 33% here.^[^ ^10^ ^]^ Something less expected is the significant increase in the energy efficiency of the ferromagnetic layer switching just by changing the Gd concentration, i.e., the different threshold fluences observed in both samples as can be seen in Table 1. Indeed, increasing the Gd concentration by 5.7% implies a threshold fluence reduction for the Co/Pt multilayer of 21.8% (from 2.11 to 1.65 mJ cm^−^²). We also note that in both cases, these threshold fluences are much smaller than the value of 11.8 mJ cm^−2^ found by Iihama et al.^[^ ^27^ ^]^ In the work by Choi and Min,^[^ ^33^ ^]^ it can be seen that increasing the Gd concentration does not change much the primary spin current. Therefore, we conclude that the Co/Pt multilayer demagnetizes the same way in both samples. We could not find a difference in the complex optical indices of both GdFeCo alloys by ellipsometry measurements, thus the laser excitation process in both samples should be the same. Consequently, we attribute this difference in threshold fluences to the secondary spin current generated by the Gd sublattice demagnetization. Because this spin current carries more angular momentum in the sample with *x* = 33%, less demagnetization is required to reverse the ferromagnetic layer.

Another interesting point is the reversal of the GdFeCo layer's magnetization in the sample with *x* = 33% with one femtosecond laser pulse. We remind the reader that under the same conditions the single GdFeCo layer does not switch. There is a simple hypothesis to understand this switching if the initial magnetic configuration is P. Assuming that conduction electrons mediate exchange scattering in AO‐HIS as in s–d models, the Co/Pt multilayer could help the magnetization reversal of the GdFeCo layer by acting as a supplementary reservoir of angular momentum. However, this explanation fails when the initial magnetic configuration is AP. It could be that the presence of the Co/Pt increases the composition range to see AOS, thanks to additional (polarized or not) hot electrons coming from this layer compared to samples without the Co/Pt multilayer. The spin current coming from the spin dependent Seebeck effect in Co/Pt multilayers^[^ ^46^ ^]^ could also play a role but this would require more careful studies and is beyond the scope of this paper. However, we note that these effects should be dependent on temperature gradients, which will be different when exciting from the top side or the bottom side, as expected from the calculated absorption profiles (see Section S6, Supporting Information).

Finally, we note that in a recent work, van Hees et al.^[^ ^28^ ^]^ used the spin current coming from a ferromagnetic layer in order to assist or hinder the AOS of a GdFeCo layer. This is a different but somewhat complementary work, which supports our conclusions. In their case, the spin current is coming from the ferromagnetic layer and changes the threshold fluence for the AOS of a Gd/Co bilayer. In our case, all threshold fluences for GdFeCo are the same, but the polarization of the spin current coming from the GdFeCo determines whether the ferromagnetic layer's magnetization switches or not. The same spin current generated by the ferromagnetic layer most likely also exists in our sample but we cannot observe its effect.

## 5 Conclusion

To conclude, we confirmed that, in a spin valve structure GdFeCo/Cu/[Co/Pt], a single femtosecond laser pulse can induce a fast change of the Gd magnetization sublattice, which generates a spin current sufficient to switch the ferromagnetic layer if the ferromagnetic layer is sufficiently demagnetized. The switching of the ferromagnetic layer is observed when the femtosecond laser induces a full magnetization switching (AO‐HIS) but also when it only induces a partial demagnetization of the GdFeCo. Indeed, we demonstrated that with a GdFeCo layer not showing AO‐HIS we could still switch the ferromagnetic layer with one single laser pulse. Moreover, we have shown that in this case the ferromagnetic layer switching requires less energy, i.e., a lower laser fluence.

This work should motivate more dynamical measurements as well as more theoretical work in order to fully understand the transfer of energy and angular momentum that seems to be the cause of this behavior and the unexpected GdFeCo single pulse switching at high Gd concentration.

## Conflict of Interest

The authors declare no conflict of interest.

## Supporting information

Supporting Information
